# Bridging the brain and gut: neuroimmune mechanisms of neuroinflammation and therapeutic insights

**DOI:** 10.3389/fncel.2025.1590002

**Published:** 2025-06-13

**Authors:** Ludmila Müller, Svetlana Di Benedetto

**Affiliations:** Center for Lifespan Psychology, Max Planck Institute for Human Development, Berlin, Germany

**Keywords:** neuroinflammation, glial cells, neurons, immune cells, cytokines, gut-brain interactions, neurodegenerative diseases, AD

## Abstract

The central nervous system (CNS) and the immune system are profoundly interconnected, engaging in a continuous dynamic exchange that regulates homeostasis, immune surveillance, and responses to injury. These interactions occur through diverse mechanisms, ranging from microglial activation and cytokine signaling to peripheral immune cell infiltration. When disrupted, this balance contributes to neurodegenerative processes, affecting cognitive function and neuronal survival. This mini-review examines the cellular and molecular foundations of neuroimmune communication, focusing on how neuroimmune interactions influence the onset and progression of neurodegenerative disorders such as Alzheimer’s disease. Key mechanisms include barrier systems, gut-brain interactions, and circadian rhythm regulation, all playing a crucial role in modulating neuroinflammatory responses. The gut-brain axis plays a pivotal role in modulating CNS function, as alterations in gut microbiota composition can trigger neuroinflammatory pathways, affect systemic immunity, and influence disease susceptibility. Both innate and adaptive immune responses are instrumental in shaping disease trajectory, highlighting the complex interplay between systemic and neural immune components. The blood-brain barrier and glymphatic system modulate immune cell trafficking and waste clearance, influencing CNS pathology. Additionally, circadian rhythm and sleep patterns regulate neuroimmune balance, with disruptions exacerbating inflammation and neurodegeneration. Neuroimmune crosstalk manifests through a spectrum of pathways, each capable of either promoting resilience or accelerating neurodegeneration. By unraveling these connections, we can gain new insights into potential strategies to modulate immune responses and restore homeostasis. This investigation underlines the necessity of integrative approaches that target immune modulation, microbiota regulation, and circadian alignment to mitigate neurodegenerative disease progression and improve therapeutic outcomes.

## 1 Introduction

The central nervous system and the immune system maintain a complex and dynamic relationship essential for regulating homeostasis, protecting against pathogens, and facilitating tissue repair. This bidirectional communication is mediated by various mechanisms, including cytokine signaling, microglial activation, and the trafficking of peripheral immune cells into the CNS. Under physiological conditions, these interactions support neuronal function and plasticity. However, when dysregulated, they can contribute to chronic neuroinflammation, a key driver of neurodegenerative diseases such as Alzheimer’s disease (AD) (Bano et al., 2024; Kwon and Koh, 2020; Matejuk et al., 2021; Müller et al., 2025; Nosi et al., 2021).

Neuroinflammation is increasingly recognized as a key pathological feature of AD, with microglial activation, blood-brain barrier (BBB) dysfunction, and systemic inflammation—all contributing to disease progression. AD is characterized by the accumulation of amyloid-beta (Aβ) plaques, tau pathology, and progressive neuronal loss (Heneka et al., 2024; Leng and Edison, 2021). Emerging evidence suggests that immune dysregulation plays a pivotal role in disease progression, with both central and peripheral immune responses influencing pathological mechanisms (Alkhalifa et al., 2023; Bano et al., 2024; Batista et al., 2024; Chachaj et al., 2023; Leng and Edison, 2021; Thakur et al., 2023).

In fact, Aβ pathology, tau aggregation, and immune activation are central to the neurodegenerative processes observed in AD. The accumulation of Aβ plaques in the brain triggers a cascade of events, beginning with the activation of microglia, the resident immune cells of the central nervous system. These microglia become reactive and release pro-inflammatory cytokines, which, in turn, exacerbate neuronal damage (Chitnis and Weiner, 2017; Kempuraj et al., 2016; Leng and Edison, 2021). Additionally, the aggregation of tau protein into neurofibrillary tangles disrupts intracellular transport and contributes to neuronal dysfunction. The interaction between Aβ, tau, and immune activation creates a vicious cycle, where inflammation promotes further tau pathology, and tau toxicity worsens inflammation, ultimately leading to neuronal death and cognitive decline (Bano et al., 2024; Chitnis and Weiner, 2017; Kang et al., 2009; Kempuraj et al., 2016; Leng and Edison, 2021).

One crucial yet often overlooked modulator of neuroimmune function is the gut-brain axis (GBA), which links the intestinal microbiota, the immune system, and neural signaling pathways (Bano et al., 2024). Disruptions in gut microbial composition, commonly referred to as dysbiosis, have been implicated in promoting systemic inflammation, altering BBB integrity, and exacerbating neurodegeneration in AD. Additionally, gut-derived neurotransmitters and vagal nerve signaling provide a direct pathway for microbial influence on brain function (Bano et al., 2024; Kalyanaraman et al., 2024; Silva et al., 2020).

In addition to the GBA, other physiological systems and barriers, such as the BBB and the glymphatic system, contribute to immune surveillance and waste clearance in the CNS (Alkhalifa et al., 2023; Caserta et al., 1998; Silva et al., 2021). These barriers regulate the exchange of immune cells and metabolites between the brain and peripheral circulation, maintaining a delicate balance between protection and pathological inflammation (Kress et al., 2014; Silva et al., 2021). Another important regulator of neuroimmune interactions is the circadian rhythm, which influences immune cell activity, BBB permeability, and metabolic function. Disruptions in circadian regulation, commonly observed in AD, further exacerbate neuroinflammatory processes, contributing to disease severity (Chachaj et al., 2023; Kadry et al., 2020; Knox et al., 2022; Szlufik et al., 2024).

This mini-review aims to explore the cellular and molecular foundations of neuroimmune communication, focusing on the interplay between the GBA, neuroinflammatory responses, and AD pathology. We will discuss the cellular and molecular mechanisms underlying gut microbiota interactions with the immune and nervous systems, the impact of BBB dysfunction on immune signaling, and the influence of circadian disruptions on neurodegeneration. Finally, we highlight potential therapeutic strategies targeting immune modulation, microbiota regulation, and circadian alignment as promising avenues for mitigating AD progression and improving therapeutic outcomes.

Recent comprehensive reviews offer in-depth coverage of broader aspects of neuroimmune interactions and neuroinflammation, providing valuable background for readers interested in a more extensive discussion (Heneka et al., 2024; Khezri and Ghasemnejad-Berenji, 2023; Seo and Holtzman, 2024). In the following section we will begin by briefly outlining the functions of barrier systems and their role in maintaining CNS homeostasis and regulating neuroinflammation.

## 2 Immune surveillance and brain barrier system

**FIGURE 1:**
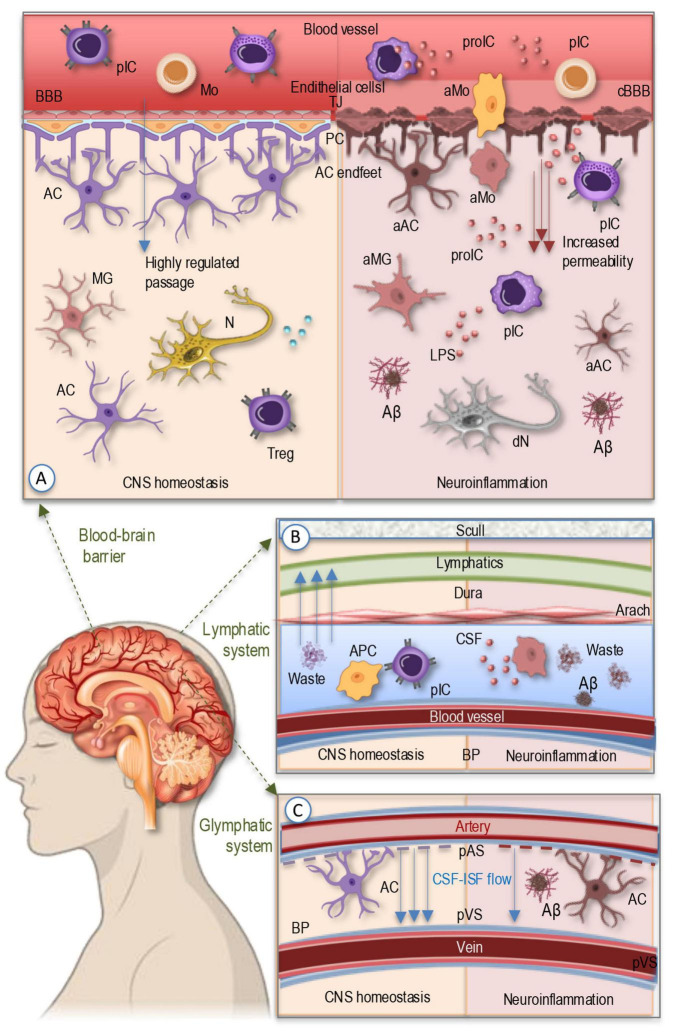
The simplified illustration of the barrier systems regulating CNS immune homeostasis and waste clearance. **(A)** BBB serves as a selective interface between the peripheral circulation and the CNS, formed by endothelial cells, tight junctions, pericytes, and astrocytic endfeet. Under homeostatic conditions, only specific peripheral immune cells, such as patrolling monocytes and subsets of T cells, can enter the CNS through controlled mechanisms. In neurodegenerative diseases like AD, chronic inflammation, oxidative stress, and endothelial dysfunction compromise BBB integrity, increasing permeability and allowing infiltration of activated monocytes and peripheral immune cells, along with pro-inflammatory cytokines, which drive neuroinflammation, impair Aβ clearance, and promote its aggregation. **(B)** The meningeal lymphatic system located within the meninges—comprising the dura, arachnoid, and pia mater—contains functional lymphatic vessels that facilitate the drainage of CNS-derived antigens and immune cells to cervical lymph nodes. In AD, impaired meningeal lymphatic function hinders antigen clearance, leading to chronic immune activation, waste aggregation, and sustained neuroinflammation. **(C)** The glymphatic system, a perivascular network regulated by CSF-ISF flow and astrocytic aquaporin channels, is responsible for clearing metabolic waste, including toxic Aβ and tau aggregates. Glymphatic dysfunction, commonly observed in aging and AD, leads to waste accumulation, exacerbating disease pathology. AC, astrocyte; aAC, activated astrocyte; Aβ, Amyloid β plaque; aIP, amyloid-like protein; aMG, activated microglia; aMo, activated monocyte; 9APC, antigen-presenting cell; BBB, blood-brain barrier; BP, brain parenchyma; cBBB, compromised blood-brain barrier; CSF, cerebrospinal fluid; dN, degenerating neuron; ISF, interstitial fluid; MG, microglia; Mo, monocytes; N, neuron; PC, pericytes; pIC, peripheral immune cells; proIC, pro-inflammatory cytokines; TJ, tight junctions; Treg, regulatory T cell.

Maintaining CNS homeostasis relies on tightly regulated mechanisms that control immune cell trafficking and prevent the infiltration of harmful substances that could trigger neuroinflammation. Among these protective mechanisms, the BBB serves as a highly selective interface between the peripheral circulation and the CNS (Kadry et al., 2020; Knox et al., 2022). Composed of endothelial cells tightly connected by junctional proteins, supported by pericytes and astrocytic endfeet, the BBB regulates the passage of molecules and immune cells into the brain (Figure 1A). Under physiological conditions, only specific immune cells, such as patrolling monocytes and certain subsets of T cells, can access the CNS through controlled mechanisms, including chemokine-mediated recruitment and adhesion molecule interactions involving vascular cell adhesion molecule-1 (VCAM-1) and intercellular adhesion molecule-1 (ICAM-1) (Huang et al., 2021; Kadry et al., 2020). However, in neurodegenerative diseases like AD, BBB integrity is compromised due to chronic inflammation, oxidative stress, and endothelial dysfunction (Alkhalifa et al., 2023). Increased permeability allows for the uncontrolled infiltration of peripheral immune cells, including activated monocytes and pro-inflammatory T cells, which exacerbate neuroinflammation and contribute to neuronal damage. Additionally, the dysregulation of efflux transporters, such as P-glycoprotein, impairs Aβ clearance, further promoting its accumulation and aggregation (Alkhalifa et al., 2023; Huang et al., 2021; Kempuraj et al., 2016; Takata et al., 2021).

Beyond the BBB, the meningeal (Figure 1B) and glymphatic systems (Figure 1C) play essential roles in immune surveillance and waste clearance (Iliff et al., 2012; Sun et al., 2018; Szlufik et al., 2024). The meninges, consisting of the dura, arachnoid, and pia mater, contain functional lymphatic vessels that facilitate the drainage of CNS-derived antigens and immune cell trafficking to cervical lymph nodes (Sun et al., 2018). This process helps regulate immune responses by enabling antigen-presenting cells to interact with peripheral immune cells, thereby maintaining a balanced immune environment (Di Benedetto et al., 2017; Louveau et al., 2018; Sun et al., 2018). In AD, meningeal lymphatic dysfunction impairs antigen clearance, leading to chronic immune activation and sustained neuroinflammation (Chachaj et al., 2023; Li et al., 2022). The glymphatic system (Figure 1C), a perivascular network driven by cerebrospinal fluid (CSF) dynamics and astrocytic aquaporin-4 (AQP4) channels, is responsible for clearing metabolic waste, including toxic protein aggregates such as Aβ and tau (Da Mesquita et al., 2018; Iliff et al., 2012). Impaired glymphatic function, often observed in aging and AD, results in the accumulation of these neurotoxic proteins, exacerbating disease pathology (Silva et al., 2021; Szlufik et al., 2024).

Disruptions in barrier integrity further amplify neuroinflammatory responses, creating a feed-forward cycle of damage (Figure 1A). Increased BBB permeability facilitates the entry of circulating pro-inflammatory cytokines, lipopolysaccharides (LPS), and immune cells into the brain parenchyma, activating microglia and astrocytes (Alkhalifa et al., 2023; Di Benedetto et al., 2017; Huang et al., 2021; Knox et al., 2022). This activation leads to the secretion of additional inflammatory mediators, such as tumor necrosis factor-alpha (TNF-α), interleukin-1 beta (IL-1β), and interleukin-6 (IL-6), which further weaken barrier function by downregulating tight junction proteins like claudin-5 and occluding (Takata et al., 2021; Versele et al., 2022). Chronic neuroinflammation disrupts homeostatic immune surveillance, skewing microglial function toward a pro-inflammatory, neurotoxic phenotype, impairing their ability to clear protein aggregates (Colonna and Butovsky, 2017; Di Benedetto et al., 2017; Kwon and Koh, 2020; Labzin et al., 2018; Müller et al., 2025) and exacerbating neurodegeneration in AD.

Thus, the combination of barrier dysfunction, impaired waste clearance, and excessive immune activation accelerates neurodegeneration, highlighting the critical interplay between barrier systems and immune regulation in AD. In the next section we will briefly introduce the main cellular and molecular players involved in neuroimmune crosstalk and their roles in maintaining CNS homeostasis and modulating immune responses.

## 3 Cellular and molecular foundation of neuroimmune communication

Neuroimmune communication is governed by a complicate multilayered network of cellular interactions and molecular signaling pathways that regulate CNS homeostasis and immune responses. The central mediators of this crosstalk include microglia, astrocytes, peripheral immune cells, and neurons, which collectively shape inflammatory and neuroprotective responses (Linnerbauer et al., 2020; Müller et al., 2025; Nosi et al., 2021; Song and Dityatev, 2018).

Microglia, the resident immune cells of the brain, are key regulators of CNS immunity. Under homeostatic conditions, they participate in synaptic pruning, debris clearance, and neuronal support (Li and Barres, 2018; Müller et al., 2025; Nosi et al., 2021). However, in AD, microglia become chronically activated, adopting a pro-inflammatory phenotype characterized by the release of cytokines, chemokines, and reactive oxygen species (ROS) (Miao et al., 2023; Valiukas et al., 2025). This sustained activation contributes to synaptic dysfunction, neuronal loss, and the exacerbation of amyloid and tau pathology (Kwon and Koh, 2020; Perry and Holmes, 2014; Wendimu and Hooks, 2022).

Astrocytes, another critical glial cell type, play a dual role in neuroimmune interactions. In healthy conditions, astrocytes maintain BBB integrity, provide metabolic support to neurons, and modulate synaptic activity. During neuroinflammation, reactive astrocytes undergo phenotypic changes, leading to either neuroprotective or neurotoxic effects (Patani et al., 2023; Sanmarco et al., 2021; Santello et al., 2019; Song and Dityatev, 2018). In AD, astrocytes can amplify inflammatory responses by releasing pro-inflammatory cytokines and promoting microglial activation, further accelerating neurodegeneration (Singh, 2022; Wu and Eisel, 2023).

Peripheral immune cells, including monocytes, macrophages, and T cells, also contribute to neuroimmune signaling. Under normal conditions, the CNS is considered an immune-privileged organ with limited immune cell infiltration. However, in AD, peripheral immune cells can cross the compromised BBB and infiltrate brain tissue, as discussed in the previous section. While some infiltrating cells may aid in Aβ clearance, others exacerbate neuroinflammation by releasing pro-inflammatory mediators (Li and Barres, 2018; Müller et al., 2025; Nosi et al., 2021).

Neurons themselves are active participants in neuroimmune communication. They express pattern recognition receptors (PRRs) that detect pathogen-associated and damage-associated molecular patterns (PAMPs and DAMPs), triggering innate immune responses (Donnelly et al., 2020; Kumar, 2019). Additionally, neuronal activity modulates glial cell function and cytokine production, influencing the overall inflammatory state of the CNS (Badimon et al., 2020; La Rosa et al., 2020; Lana et al., 2016; Lim et al., 2013; Müller et al., 2025; Song and Dityatev, 2018).

Cytokines, chemokines, and neurotransmitters serve as molecular messengers that orchestrate neuroimmune interactions. Pro-inflammatory cytokines, such as TNF-α, IL-6, and IL-1β, are upregulated in AD and drive microglial and astrocyte activation, synaptic dysfunction, and neuronal apoptosis (Abdelhamed et al., 2025; Morimoto et al., 2011). In contrast, anti-inflammatory cytokines, including interleukin-10 (IL-10) and transforming growth factor-beta (TGF-β), play a counter-regulatory role by dampening excessive inflammation and promoting tissue repair (Alboni and Maggi, 2015; Leng and Edison, 2021; Prieto and Cotman, 2017; Schneider et al., 1998).

Chemokines, such as C-C motif chemokine ligand 2 (CCL2) and C-X-C motif chemokine ligand 10 (CXCL10), coordinate the migration and activation of immune cells within the CNS (Reaux-Le Goazigo et al., 2013; Williams et al., 2014). In AD, increased chemokine signaling promotes peripheral immune cell infiltration and sustained microglial activation, exacerbating neuroinflammation. Dysregulation of chemokine pathways contributes to neurodegeneration by recruiting immune cells that enhance pro-inflammatory cascades (Clarner et al., 2015; Koper et al., 2018; Pawelec et al., 2020; Thakur et al., 2023).

Neurotransmitters also influence neuroimmune interactions by modulating glial cell activity and immune responses. For example, glutamate, the primary excitatory neurotransmitter, can induce excitotoxicity and trigger inflammatory responses when its levels are dysregulated. Acetylcholine, known for its role in cognition, also exerts anti-inflammatory effects by suppressing pro-inflammatory cytokine release (Lovinger, 2008; Müller et al., 2025; Teleanu et al., 2022b). Dysfunctions in cholinergic signaling, which are characteristic of AD, may further amplify neuroinflammation and contribute to disease progression (Chen et al., 2022). The balance between these molecular signals is critical for maintaining homeostasis, and its disruption in AD leads to chronic neuroinflammation and neurodegeneration (Teleanu et al., 2022b; Thakur et al., 2023).

Overall, the neuroimmune crosstalk in AD is a multifaceted process involving microglia, astrocytes, peripheral immune cells, and neurons, all of which contribute to disease progression through complex signaling networks. Understanding the intricate interplay among these cellular and molecular participants provides a foundation for developing targeted therapeutic strategies to mitigate neuroinflammation and enhance neuroprotection. In the next section we will examine the modulatory role of the gut-brain axis, which integrates the intestinal microbiota, the immune system, and neural signaling pathways.

## 4 The gut-brain axis and neuroinflammation

**FIGURE 2:**
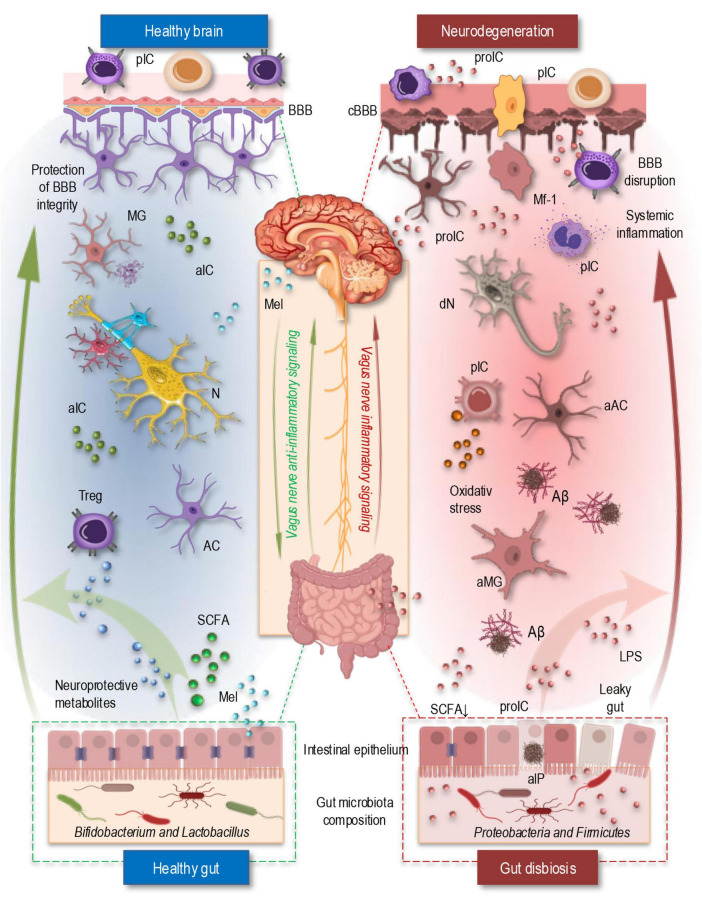
The simplified illustration of the gut-brain axis in a healthy state (left) and under neurodegenerative conditions in AD (right). In the healthy brain (top left, blue area), an intact BBB regulates immune cell trafficking and prevents excessive neuroinflammation. Microglia remain in a homeostatic state, and neuroprotective metabolites and SCFAs support neuronal function and anti-inflammatory responses. The vagus nerve enables bidirectional communication between the gut and brain that help maintain neuronal homeostasis. Additionally, melatonin, primarily produced by the pineal gland and also synthesized in the gut, regulates circadian rhythms, enhances BBB integrity, and exerts antioxidant and anti-inflammatory effects, contributing to neuronal protection. In the healthy gut (bottom left, blue area), a balanced microbiota composition supports gut barrier integrity, regulate immune response through Tregs, and produces SCFAs that modulate neuroimmune interactions. In contrast, during neurodegeneration (top right, red area), a compromised BBB allows infiltration of peripheral immune cells and pro-inflammatory cytokines, leading to glial cell activation, oxidative stress, and Aβ-plaque formation. The vagus nerve’s regulatory function is impaired, supporting inflammatory signaling and exacerbating neuroinflammation. Simultaneously, gut dysbiosis (bottom right, red area) increases intestinal permeability, allowing bacterial endotoxins like LPS to enter circulation, triggering systemic inflammation and BBB disruption. Dysbiotic microbes also release amyloid-like proteins, promoting cross-seeding with brain-derived Aβ and further driving neurodegeneration. Aβ, Amyloid β plaque; aAC, activated astrocyte; aIC, anti-inflammatory cytokines; aIP, amyloid-like protein; aMG, activated microglia; AC, astrocyte; BBB, blood-brain barrier; cBBB, compromised blood-brain barrier; dN, degenerating neuron; LPS, lipopolysaccharide; Mel, melatonin; MG, microglia; Mf-1, macrophage type 1; N, neuron; pIC, peripheral immune cells; proIC, pro-inflammatory cytokines; SCFA, short-chain fatty acids; Treg, regulatory T cell.

The gut-brain axis is an essential bidirectional communication system that links the gastrointestinal tract and the CNS (Figure 2), integrating neural, endocrine, immune, and metabolic signaling pathways (Erny et al., 2015; Lin et al., 2019). These complex multilayered interactions play a crucial role in maintaining homeostasis and modulating neuroinflammatory processes. The gut microbiota, a diverse community of microorganisms residing in the intestine, significantly influences CNS function by modulating systemic immune responses, producing bioactive metabolites, and interacting with neural pathways (Figure 2, left). Emerging evidence suggests that disruptions in the gut microbiota composition, known as dysbiosis, have been implicated in neurodegenerative disorders, including AD, by promoting systemic inflammation and altering neuroimmune homeostasis (Figure 2, right; Bano et al., 2024; Krishaa et al., 2023; Lin et al., 2019).

### 4.1 Gut microbiota alterations in AD

Alterations in the gut microbiota have been extensively studied in AD, uncovering several key changes that likely contribute to the disease’s progression (Bano et al., 2024; Collins et al., 2012). A significant finding is the reduction in beneficial bacteria, such as *Bifidobacterium* and *Lactobacillus*, which are essential for maintaining gut barrier integrity and promoting the production of neuroprotective metabolites. These microbes play a crucial role in modulating the gut immune system and supporting neuronal health (Figure 2, left). Their depletion may disrupt the gut-brain axis, compromising both the gut barrier and the brain’s defense mechanisms, thus facilitating neurodegenerative processes linked to AD (Bano et al., 2024; Shabbir et al., 2021).

In contrast, an increase in pro-inflammatory bacterial species, particularly within the phyla *Proteobacteria* and *Firmicutes*, has been observed in individuals with AD. These bacteria are known to produce endotoxins such as LPS, which can induce systemic inflammation (Figure 2, riht). Chronic, low-grade inflammation is a well-established factor in AD, contributing to blood-brain barrier disruption, oxidative stress, and microglial activation. This inflammatory cascade accelerates neurodegeneration and promotes the progression of AD pathology, highlighting the importance of microbial imbalances in driving disease mechanisms (Bano et al., 2024; Collins et al., 2012; Lin et al., 2019; Shabbir et al., 2021).

Another emerging area of research is the bacterial amyloids and cross-seeding hypothesis (Subedi et al., 2022). Some gut bacteria are capable of producing amyloid-like proteins that share structural similarities with amyloid-β found in the brains of AD patients. This phenomenon, known as “cross-seeding,” suggests that bacterial amyloids may interact with brain-derived Aβ, promoting plaque formation and exacerbating neuroinflammation (Blanco-Miguez et al., 2021; Fernandez-Calvet et al., 2024; Javed et al., 2020). Such interactions potentially create a feedback loop that amplifies the pathological processes of AD, linking the gut microbiota to brain amyloid aggregation and neurodegeneration (Chaudhuri et al., 2019; Friedland and Chapman, 2017; Kohler et al., 2016; Subedi et al., 2022).

The disruption of the gut-brain axis is essential aspect of gut microbiota alterations in AD. Dysbiosis, or microbial imbalance, can lead to increased intestinal permeability, commonly referred to as “leaky gut,” allowing harmful bacteria and their by-products, such as LPS, to enter the bloodstream (Heston et al., 2023). This systemic inflammation can affect the central nervous system, intensifying neuroinflammation and neurodegeneration in AD (Kohler et al., 2016).

Multiple studies suggest that gut barrier integrity is compromised in Alzheimer’s disease (Boschetti et al., 2023; He et al., 2023). In patients with AD and amnestic mild cognitive impairment (aMCI), elevated serum levels of zonulin—a regulator of intestinal tight junctions—indicate increased gut permeability, with higher levels predicting progression from aMCI to AD (Boschetti et al., 2023). Experimental studies in transgenic mouse models of AD further support these findings, showing increased intestinal permeability, reduced expression of tight junction proteins such as occludin and claudin-1, and accumulation of amyloid-beta (Aβ) in the intestinal epithelium (He et al., 2023). These observations suggest that gut barrier dysfunction may contribute to systemic inflammation and neurodegenerative processes in AD.

These findings underscore the complex relationship between gut health and AD, suggesting that microbial modulation could be a promising avenue for therapeutic intervention in the disease. Understanding the mechanisms and signaling pathways linking dysbiosis to neuroinflammation could open new possibilities for restoring a healthy microbial balance to mitigate the effects of neurodegeneration.

### 4.2 Mechanisms linking the gut microbiota to neuroinflammation and AD

The connection between the gut microbiota and AD pathology is complex, involving a range of biochemical signaling pathways that significantly impact neuroinflammation, neuronal dysfunction, and the progression of the disease. A key factor in these processes is the production of microbial metabolites, such as short-chain fatty acids (SCFAs) and tryptophan-derived metabolites (Kalyanaraman et al., 2024; Silva et al., 2020). SCFAs, including acetate, propionate, and butyrate, are produced by gut bacteria during the fermentation of dietary fibers. These metabolites can influence the BBB by regulating tight junction protein expression and enhancing endothelial cell integrity (Chenghan et al., 2025). In AD, reduced SCFA levels impair microglial function by shifting microglial polarization toward a pro-inflammatory phenotype. This shift is driven by altered signaling pathways, such as the inhibition of histone deacetylases (HDACs) and activation of the NF-κB pathway, both of which promote the expression of inflammatory cytokines. The resulting chronic neuroinflammation disrupts synaptic plasticity and accelerates neuronal damage, contributing to the progression of AD (Doifode et al., 2021; Krishaa et al., 2023; Lin et al., 2019; Thakur et al., 2023).

Cytokine signaling plays a central role in the gut-brain communication in AD. Dysbiosis in AD leads to an imbalance in the gut microbiota, which triggers the release of pro-inflammatory cytokines like IL-6, TNF-α, and IL-1β from immune cells (Krishaa et al., 2023; Lin et al., 2019). These cytokines are capable of crossing the BBB via active transport mechanisms involving endothelial cell receptors such as ICAM-1 and VCAM-1. Once in the brain, these cytokines activate microglia through their respective receptors, such as TNFR1 and IL-1R, leading to the activation of downstream signaling pathways like the JAK/STAT pathway, which further promotes the production of inflammatory mediators (Valiukas et al., 2025). This process triggers tau hyperphosphorylation and facilitates Aβ aggregation through the enhanced activation of glycogen synthase kinase 3β (GSK-3β) and β-secretase (BACE1), which contribute to the hallmark neurofibrillary tangles and plaques found in AD brains (Krishaa et al., 2023; Kumari et al., 2023; Leng and Edison, 2021; Lian et al., 2016; Mayne et al., 2020; Santello et al., 2019; Teleanu et al., 2022a; Tilleux and Hermans, 2007; Wu and Eisel, 2023).

The *vagus nerve* also plays a significant role in mediating gut-brain interactions, particularly in the regulation of neuroinflammation. The *vagus nerve* transmits afferent signals from the gut to the brain and modulates the activation of immune responses through the cholinergic anti-inflammatory pathway (Figure 2). Acetylcholine (ACh), released by vagal fibers, binds to α7 nicotinic acetylcholine receptors (α7nAChR) on immune cells, particularly macrophages and microglia. This binding inhibits the release of pro-inflammatory cytokines through the NF-κB signaling pathway, thereby limiting neuroinflammation (Bonaz et al., 2018; Decarie-Spain et al., 2024; Onimus et al., 2024). In AD, however, altered vagal tone impairs this regulatory pathway, leading to a failure to suppress excessive inflammatory responses. This dysfunction results in sustained neuroinflammation, which accelerates neuronal loss and cognitive decline in AD patients (Bano et al., 2024).

LPS, that are components of the outer membrane of Gram-negative bacteria, are another crucial mediator in gut-brain signaling. When the gut barrier is compromised due to dysbiosis or inflammation, LPS can translocate from the intestines into the bloodstream. Once in the systemic circulation, LPS activates the immune system by binding to Toll-like receptor 4 (TLR4) on immune cells, including microglia and macrophages. This binding triggers the activation of the myeloid differentiation primary response gene 88 (MyD88)-dependent pathway, leading to the production of pro-inflammatory cytokines. These cytokines can cross the BBB and activate neuroinflammatory pathways in the brain. Elevated LPS levels have been shown to correlate with BBB disruption, microglial activation, and increased oxidative stress, all of which contribute to the pathophysiology of AD. The persistent activation of these pathways by LPS exacerbates the inflammatory environment in the brain, leading to further neuronal injury and cognitive impairment (Doifode et al., 2021; Paudel et al., 2020; Peng et al., 2021; Yang et al., 2020).

Together, these mechanisms demonstrate how gut-derived signals—ranging from microbial metabolites to immune mediators like cytokines and LPS—converge on key cellular processes that drive the neuroinflammatory and neurodegenerative aspects of AD. These pathways provide a detailed framework for understanding the complex interactions between the gut microbiota and AD pathology. Additionally, gut microbiota composition follows circadian fluctuations, and dysbiosis resulting from disrupted sleep can amplify neuroinflammatory pathways via microbial metabolites and immune signaling cascades. The next section examines how disruptions in circadian rhythms and sleep patterns can disturb gut-brain interactions, exacerbating neuroinflammation and contributing to the progression of neurodegenerative diseases.

## 5 Circadian rhythm, sleep, and immune modulation

The interplay between circadian rhythms, sleep, and immune modulation plays a crucial role in maintaining neuroimmune homeostasis, with significant implications for neurodegenerative diseases such as AD. Circadian rhythms, governed by the central clock in the suprachiasmatic nucleus (SCN) and peripheral clocks in various tissues, regulate immune cell activity, cytokine secretion, and neuroinflammatory responses (Kang et al., 2009; Whittaker et al., 2023). Microglia, the primary immune cells of the brain, exhibit circadian fluctuations in their surveillance and inflammatory profiles, which are driven by clock genes such as *BMAL1*, *CLOCK*, *PER*, and *CRY* (Fonken et al., 2015; Wang and Li, 2021; Wang et al., 2020). Disruptions in these rhythms can lead to an imbalance in microglial function, shifting them toward a pro-inflammatory phenotype that contributes to chronic neuroinflammation, a key driver of AD pathology (Abe et al., 2022; Musiek and Holtzman, 2016; Nassan and Videnovic, 2022; Niu et al., 2022).

Sleep is essential for modulating immune responses and clearing neurotoxic waste products from the brain. The glymphatic system, which facilitates the removal of metabolic byproducts such as Aβ and tau, is most active during deep sleep. Sleep deprivation or fragmented sleep patterns impair glymphatic clearance, leading to the accumulation of Aβ and tau aggregates, which are hallmarks of AD. Moreover, sleep disturbances alter the secretion of pro- and anti-inflammatory cytokines, increasing levels of IL-6, TNF-α, and IL-1β (Vasciaveo et al., 2023; Xie et al., 2013). This heightened inflammatory state further exacerbates neurodegeneration by promoting oxidative stress, BBB dysfunction, and synaptic dysregulation (Abe et al., 2022; Musiek and Holtzman, 2016; Niu et al., 2022). Sleep disruption exerts widespread effects on immune regulation, not only altering peripheral immune cell profiles but also promoting microglial activation and impairing astrocytic phagocytosis. Together, these changes foster a pro-inflammatory milieu within the CNS, which may exacerbate neurodegenerative processes (Bellesi et al., 2017; Hurtado-Alvarado et al., 2016; Wisor et al., 2011).

Chronic circadian misalignment, often observed in aging and neurodegenerative conditions, weakens the immune system’s ability to regulate inflammation effectively. In AD, altered sleep-wake cycles and reduced melatonin production contribute to increased neuronal excitotoxicity, mitochondrial dysfunction, and impaired cellular repair mechanisms. Melatonin, a hormone primarily produced by the pineal gland and also by gut enterochromaffin cells—specialized epithelial cells involved in neurotransmitter and hormone release— plays a crucial role in gut-brain communication (Chen et al., 2011; Chen et al., 2024; Zhang et al., 2025). It reinforces circadian rhythms, strengthens gut and BBB integrity, and exerts potent anti-inflammatory effects, thereby contributing to neuronal protection. Melatonin plays a crucial role in regulating circadian rhythms and maintaining sleep-wake cycles. Its secretion follows a diurnal pattern, rising in the evening to promote sleep and decreasing in the morning to support wakefulness (Musiek and Holtzman, 2016; Nassan and Videnovic, 2022; Zisapel, 2018).

Beyond its function as a sleep regulator, melatonin exerts neuroprotective effects through its antioxidant and mitochondrial-stabilizing properties. It scavenges ROS, reduces oxidative stress, and modulates the activity of inflammatory pathways, including NF-κB signaling, which is often dysregulated in neurodegenerative diseases. Additionally, melatonin influences synaptic plasticity and enhances the clearance of toxic protein aggregates such as Aβ through its interactions with the glymphatic system (Hardeland et al., 1993; Reiter, 1995; Roy et al., 2021; Veneroso et al., 2009). In AD and other neurodegenerative conditions, melatonin levels are often diminished, contributing to circadian misalignment, increased neuroinflammation, and impaired cognitive function (Steinbach and Denburg, 2024; Sumsuzzman et al., 2021; Zisapel, 2018).

Thus, the bidirectional relationship between sleep disturbances and neuroinflammation creates a self-perpetuating cycle that accelerates neurodegeneration, with reduced melatonin levels further exacerbating circadian misalignment and oxidative stress. This underscores the need for therapeutic strategies that restore circadian balance, enhance melatonin signaling, and improve sleep quality to mitigate neuroinflammation and neurodegeneration.

## 6 Therapeutic perspectives by modulating neuroinflammation

Targeting neuroinflammation has emerged as a promising therapeutic strategy for AD, given its critical role in disease progression. Various approaches aim to modulate immune responses, restore homeostasis, and mitigate the detrimental effects of chronic inflammation on neuronal function. One key avenue involves repurposing anti-inflammatory drugs, such as non-steroidal anti-inflammatory drugs (NSAIDs) and corticosteroids, which have been investigated for their ability to reduce microglial activation and cytokine release. However, clinical trials have yielded mixed results, likely due to the timing of intervention, as inflammation may have both protective and detrimental effects at different disease stages (Ajmone-Cat et al., 2010; Dhapola et al., 2021).

Several important approaches involve regulating microglial activation states. Modulating microglial function through colony-stimulating factor 1 receptor (CSF1R) inhibitors, such as PLX3397, has been shown to shift microglia toward a homeostatic phenotype, reducing neuroinflammation and cognitive decline in preclinical models (Dagher et al., 2015; Son et al., 2020; Sosna et al., 2018). PLX3397 acts by blocking CSF1R signaling, which is essential for microglial survival and proliferation, thereby reducing pathogenic microglial populations.

Additionally, small molecules targeting toll-like receptors (TLRs), such as TLR4 antagonists, aim to limit microglial overactivation and excessive cytokine release. TLR4 antagonists inhibit NF-κB signaling pathways that drive pro-inflammatory cytokine production. The STAT3 signaling pathway also plays a crucial role in astrocyte reactivity, and its inhibition has been explored as a means to restore astrocyte homeostasis (Cui et al., 2020; Islam et al., 2025). Furthermore, boosting astrocytic glutamate uptake through upregulation of excitatory amino acid transporters (EAATs) can help prevent excitotoxicity, a process that exacerbates neuronal damage in AD (Paudel et al., 2020; Yang et al., 2020; Zhang et al., 2019).

Beyond glial cells, peripheral immune modulation has gained increasing attention. Strategies to modulate peripheral immune responses include the use of regulatory T-cell (Treg) therapies. Tregs are specialized lymphocytes that help suppress excessive immune activation and maintain immune homeostasis (Liston et al., 2022). Tregs exert their anti-inflammatory effects through secretion of inhibitory cytokines (e.g., IL-10, TGF-β) and direct suppression of effector T-cell proliferation. By dampening neuroinflammatory responses while preserving essential immune surveillance, Tregs offer a promising therapeutic target. Certain immunomodulatory drugs, such as fingolimod, enhance Treg function and have shown potential in reducing neuroinflammation in AD models (Angelopoulou and Piperi, 2019). Fingolimod acts as a sphingosine-1-phosphate receptor modulator, sequestering lymphocytes in lymph nodes and thereby reducing CNS infiltration.

Cytokine-targeting therapies are also being explored to dampen neuroinflammation in AD. Monoclonal antibodies against TNF-α (e.g., infliximab) and IL-1β (e.g., canakinumab) have been investigated for their ability to reduce chronic inflammation and protect neuronal integrity. These therapies neutralize key pro-inflammatory cytokines that amplify microglial and astrocytic activation. While systemic immunosuppression poses risks, localized delivery methods, such as nanoparticle-based cytokine inhibitors, may provide a more targeted approach with fewer side effects (Costagliola et al., 2022; Dhapola et al., 2021).

Another emerging strategy involves targeting metabolic and cellular stress responses implicated in AD. Enhancing autophagy, the cellular process responsible for clearing damaged proteins and organelles, has shown promise in mitigating neuroinflammation and preventing Aβ accumulation. Pharmacological activators of autophagy, such as rapamycin and spermidine, are being explored for their potential neuroprotective effects (Liu et al., 2022; Yang and Zhang, 2020). Rapamycin inhibits mTOR signaling, thereby promoting autophagy and reducing protein aggregates and inflammatory responses. Additionally, promoting mitochondrial health through compounds like nicotinamide adenine dinucleotide (NAD+) precursors or coenzyme Q10 may help counteract oxidative stress and neuroinflammatory damage (Mantle and Hargreaves, 2022; Sangineto et al., 2023).

Resolution of inflammation represents a complementary strategy, focusing on enhancing endogenous mechanisms that terminate neuroinflammatory responses. Specialized pro-resolving lipid mediators (SPMs), including resolvins, protectins, and maresins, are derived from omega-3 fatty acids and actively promote inflammation resolution, neuronal repair, and microglial phagocytosis of toxic proteins (Hong and Lu, 2013). SPMs bind to specific G-protein-coupled receptors, inhibiting pro-inflammatory signaling pathways and stimulating tissue repair mechanisms. Therapeutic supplementation with SPMs or their synthetic analogs offers a promising avenue for restoring immune balance in AD (Doyle et al., 2018; Farooqui, 2012). In addition to their anti-inflammatory properties, these pro-resolving lipid mediators support gut barrier integrity and play a key role in regulating the gut-brain axis. The next section will explore potential strategies for restoring gut microbiota balance and mitigating neuroinflammatory processes in AD.

## 7 Strategies aimed at restoring gut microbiota balance

Given the emerging role of the gut-brain axis in neuroinflammation and AD pathology, several therapeutic strategies have been proposed to restore gut microbiota balance and mitigate inflammatory processes. These approaches focus on modulating microbial composition, enhancing beneficial metabolite production, and reducing gut permeability, which in turn influences neuroimmune signaling and brain function.

Probiotic and prebiotic supplementation with beneficial bacterial strains, such as *Bifidobacterium* and *Lactobacillus*, has been explored for its potential to modulate immune responses and restore microbial homeostasis (Mazziotta et al., 2023). These bacteria produce key metabolites, including SCFAs and tryptophan derivatives, which regulate microglial activation and blood-brain barrier integrity (Gao et al., 2020). *Bifidobacterium* species promote anti-inflammatory responses by increasing IL-10 production and decreasing pro-inflammatory cytokines like IL-6 and TNF-α (Azad et al., 2018). Additionally, probiotics can enhance gut barrier function by strengthening tight junction proteins, reducing systemic inflammation and endotoxin leakage (Gou et al., 2022). Prebiotics, such as inulin and fructooligosaccharides, support the growth of beneficial bacteria by providing fermentable substrates that enhance SCFA production and modulate inflammatory signaling (Pluta et al., 2020; Zhang et al., 2023).

Probiotic supplementation has been tested in Alzheimer’s disease models, showing promising effects on cognitive function, amyloid-beta accumulation, and neuroinflammation. For example, *Bifidobacterium breve* MCC1274 improved memory and reduced hippocampal Aβ levels in APP knock-in mice by promoting non-amyloidogenic processing of APP (Jung et al., 2021). Similarly, *Lactobacillus plantarum* KY1032 and *Lactobacillus curvatus* HY7601 improved memory and reduced neuroinflammation in 3xTg-AD mice (Medeiros et al., 2024). These probiotics modulate gut-derived inflammatory pathways and enhance gut-brain communication by increasing the production of neuroprotective metabolites. These results suggest probiotics may be a potential therapeutic strategy for AD.

Postbiotic and metabolite-based therapies also hold great promise. In addition to live bacteria, microbial-derived metabolites such as SCFAs, indoles, and secondary bile acids play a key role in immune modulation and neuroprotection. SCFAs, including butyrate, propionate, and acetate, regulate inflammation by inhibiting histone deacetylases (HDACs), activating G-protein-coupled receptors (GPCRs), and supporting regulatory T-cell function. Butyrate, in particular, enhances the integrity of the gut epithelium, reducing gut-derived endotoxin leakage and systemic inflammation. Butyrate strengthens tight junctions in the intestinal epithelium and inhibits NF-κB-mediated inflammatory signaling pathways. Tryptophan metabolites, including indole derivatives, interact with the aryl hydrocarbon receptor (AhR) to modulate microglial activation and astrocyte function, presenting a potential therapeutic avenue for AD (Pluta et al., 2020; Zhang et al., 2023).

Fecal Microbiota Transplantation (FMT)—the transfer of gut microbiota from a healthy donor to a recipient, has gained interest as a potential intervention for restoring microbial balance in neurological disorders (Matheson and Holsinger, 2023). While primarily used to treat conditions like *Clostridioides* difficile infection, preclinical studies suggest that FMT may modulate neuroinflammation by replenishing beneficial bacteria and restoring metabolic homeostasis. FMT has been shown to reduce gut permeability, decrease systemic endotoxin levels, and shift microglial activation toward a homeostatic phenotype in AD models. Experimental models of AD have shown improvements in cognitive function and reduced neuroinflammatory markers following FMT, though clinical applications remain in early stages (Nassar et al., 2022; Vendrik et al., 2020).

Diet plays a crucial role in shaping gut microbiota composition and influencing systemic inflammation. A Mediterranean diet, rich in polyphenols, fiber, and omega-3 fatty acids, has been associated with increased microbial diversity and reduced neuroinflammatory markers in AD (Nagpal et al., 2019). Polyphenols enhance the growth of beneficial gut bacteria and inhibit the production of LPS, a potent endotoxin that triggers systemic inflammation. Polyphenols found in fruits, vegetables, and green tea exert anti-inflammatory and antioxidant effects by modulating microbial metabolism and inhibiting pro-inflammatory cytokine production. Omega-3 fatty acids, particularly docosahexaenoic acid (DHA), support gut barrier integrity and promote the production of anti-inflammatory lipid mediators such as resolvins and protectins (Doyle et al., 2018; Farooqui, 2012; Weinreb et al., 2004).

Additionally, novel therapeutic approaches aim to manipulate the microbiome through pharmacological interventions. Antibiotics have been investigated for their effects on gut microbial composition and neuroinflammation, though their broad-spectrum activity may lead to unintended dysbiosis. Selective antibiotics or narrow-spectrum agents aim to eliminate specific pro-inflammatory bacterial strains while preserving overall microbial diversity. More targeted strategies, such as bacteriophage therapy, seek to selectively reduce pro-inflammatory bacterial populations while preserving beneficial microbes. Additionally, microbial enzyme inhibitors, such as those targeting bacterial amyloid production, could help prevent cross-seeding of gut-derived amyloids with brain Aβ, reducing aggregation and neurotoxicity (Chaudhuri et al., 2019; Sohrab et al., 2014; Subedi et al., 2022).

As discussed earlier, emerging evidence suggests that circadian rhythms influence gut microbiota composition and function, with disruptions in sleep-wake cycles contributing to dysbiosis and increased neuroinflammation. Interventions aimed at restoring circadian balance, including light therapy, time-restricted eating, and melatonin supplementation, may help regulate microbial rhythms and improve gut-brain communication. Melatonin not only regulates sleep but also exhibits anti-inflammatory effects by modulating gut microbiota composition and suppressing oxidative stress. Physical activity has also been shown to promote microbial diversity and SCFA production, supporting anti-inflammatory pathways relevant to AD progression (Whittaker et al., 2023; Xie et al., 2022; Zang et al., 2023).

Together, these strategies offer a multifaceted approach to restoring gut microbiota balance and mitigating neuroinflammatory processes in AD. While many interventions remain in experimental stages, growing evidence supports the potential of microbiome-targeted therapies as adjunctive treatments for neurodegenerative diseases. Given the complexity of neuro-immune interactions in AD, a multi-targeted therapeutic approach may be necessary. Combining anti-inflammatory, immune-modulating, and neuroprotective strategies could provide synergistic benefits, offering a more effective means of slowing disease progression. Precision medicine strategies, incorporating genetic, metabolic, and microbiome profiling, could help tailor treatments to individual patients, optimizing efficacy while minimizing side effects.

## 8 Conclusion

Neuroinflammation and disrupted neuroimmune interactions play a crucial role in driving neurodegeneration, contributing to neuronal dysfunction, synaptic loss, and disease progression. Growing evidence highlights the decisive role of the gut-brain axis in neuroinflammation and AD pathology. Dysbiosis, microbial metabolites, and immune signaling collectively influence neuroimmune responses, contributing to disease progression. Key mechanisms include microglial and astrocyte activation, cytokine-mediated inflammation, and disruptions in the blood-brain barrier.

Therapeutic strategies targeting inflammatory immune modulation, gut microbiota regulation, and metabolic interventions offer promising avenues for mitigating neuroinflammation in AD. Approaches such as postbiotics, SCFA-based therapies, and time-restricted feeding may help restore gut homeostasis and systemic immune balance.

Future research should focus on identifying specific microbial signatures linked to AD progression, optimizing microbiota-targeted therapies, and exploring personalized treatment approaches. Additionally, integrating multi-omics techniques, including metabolomics and transcriptomics, could provide deeper insights into gut-brain interactions, paving the way for novel biomarker discovery and precision medicine strategies.
